# Clinical Significance of Plasma CD9-Positive Exosomes in HIV Seronegative and Seropositive Lung Cancer Patients

**DOI:** 10.3390/cancers13205193

**Published:** 2021-10-16

**Authors:** Foteinos-Ioannis Dimitrakopoulos, Anastasia E. Kottorou, Kristen Rodgers, John Timothy Sherwood, Georgia-Angeliki Koliou, Beverly Lee, Andrew Yang, Julie Renee Brahmer, Stephen B. Baylin, Stephen C. Yang, Hajime Orita, Alicia Hulbert, Malcolm V. Brock

**Affiliations:** 1 Division of Thoracic Surgery, Department of Surgery, Johns Hopkins University School of Medicine, Baltimore, MD 21287, USA; fodimitrakopoulos@upatras.gr (F.-I.D.); kottorou@upatras.gr (A.E.K.); kpelosk1@jhmi.edu (K.R.); blee55@jhmi.edu (B.L.); ahyang200@gmail.com (A.Y.); syang7@jhmi.edu (S.C.Y.); 2 Molecular Oncology Laboratory, Division of Oncology, Department of Internal Medicine, Medical School, University of Patras, 26504 Patras, Greece; 3 Mary Washington Hospital Center, Fredericksburg, VA 22401, USA; Tim.Sherwood@mwhc.com; 4 Section of Biostatistics, Data Office, Hellenic Cooperative Oncology Group (HECOG), 11526 Athens, Greece; g_koliou@hecog.ondsl.gr; 5 Department of Oncology, Johns Hopkins School of Medicine, Baltimore, MD 21231, USA; brahmju@jhmi.edu; 6 Department of Oncology, Sidney Kimmel Comprehensive Cancer Center at Johns Hopkins Bayview, Johns Hopkins University, Baltimore, MD 21231, USA; sbaylin@jhmi.edu; 7 Department of Gastroenterology and Minimally Invasive Surgery, Juntendo University Hospital, Tokyo 113-8421, Japan; oriori@juntendo.ac.jp; 8 Department of Surgery, The University of Illinois at Chicago, College of Medicine, Chicago, IL 60612, USA

**Keywords:** exosomes, lung cancer, prognosis, CD9, HIV, NSCLC, SCLC

## Abstract

**Simple Summary:**

The role of exosomes in HIV (human immunodeficiency virus) as well as in cancer patients seems to be pivotal. The aim of our retrospective study was to assess the potential clinical value of CD9-positive plasma exosomes in lung cancer patients, patients with lung granulomas, healthy individuals, and HIV-positive patients with or without lung cancer. This study shows that CD9-positive plasma exosome concentrations differ between healthy controls, patients with immunocompetent pulmonary granulomas and patients with lung cancer. In addition, CD9-positive plasma exosomes are increased in HIV seropositive and HIV seronegative lung cancer patients compared to healthy controls, while chemotherapy-treated lung cancer patients have lower plasma exosome levels. This study also shows that in chemotherapy-naïve patients, plasma exosome levels are directly correlated with a prognosis with higher concentrations being associated with a longer, overall survival. These findings further support previous literature on the translational significance of total plasma exosomes in cancer patients, despite different immunological contexts.

**Abstract:**

Recently, the role of exosomes in the progression of both cancer and HIV (human immunodeficiency virus) has been described. This study investigates the clinical significance of CD9-positive plasma exosomes in lung cancer patients, healthy individuals, and HIV-positive patients with or without lung cancer. Using a verified with transmission electron microscopy double-sandwich ELISA technique, plasma-derived exosomes were isolated and quantified from 210 lung cancer patients (including 44 metastatic patients with progressive disease after chemotherapy), 49 healthy controls, 20 patients with pulmonary granulomas, 19 HIV+ patients with lung cancer, 31 HIV+ patients without cancer, and 3 HIV+ patients with pulmonary granulomas. Plasma exosome concentrations differed between healthy controls, patients with immunocompetent pulmonary granulomas and patients with lung cancer even after chemotherapy (*p* < 0.001). Lung cancer patients after chemotherapy had lower exosome concentrations compared to patients with untreated lung cancer or granuloma (*p* < 0.001 for both). HIV+ patients without lung cancer had significantly higher exosome concentrations compared to HIV+ patients with lung cancer (*p* = 0.016). Although exosome concentrations differed between all different lung cancer histologies and healthy controls (*p* < 0.001 for all histologies), adjusted statistical significance was oµy retained for patients with granulomas and SCLC (Small-cell lung cancer, *p* < 0.001). HIV-induced immunodeficient patients with or without lung cancer had lower plasma exosomes compared to immunocompetent granuloma and lung cancer patients (*p* < 0.001). Finally, higher plasma exosomes were associated both on univariate (*p* = 0.044), and multivariate analysis (*p* = 0.040) with a better 3-year survival in stage II and III NSCLC (Non-small-cell lung carcinoma) patients. In conclusion, our study shows that CD9-positive plasma exosomes are associated with both lung cancer and HIV, prior chemotherapy, as well as with survival, suggesting a possible prognostic value.

## 1. Introduction

Lung cancer remains the leading cause of cancer-related deaths worldwide. Although genetic and epigenetic knowledge of lung cancer pathobiology has impressively expanded over the years, only recently has there been substantial progress clinically with the implementation of immunotherapeutic agents and specific kinase inhibitors [1,2]. Importantly absent have been concomitant discoveries in predictive biomarkers of treatment, a gap that could be bridged with exosomes [3].

Exosomes are virus-like extracellular vesicles (EV) energetically produced from all cells through a sophisticated, and as yet modified endocytic procedure, which leads to the exocytosis of these nanoscale intercellular message carriers [4]. In the last five years especially, there has been a growing number of publications shedding light on the role of exosomes and their cargo in cancer pathogenesis [5]. Although little is known about the role of primary-tumor-derived exosomes in lung cancer progression, their importance in determining organ-specific cancer metastasis by binding to corresponding matrix proteins in a distant organ has obvious clinical relevance [6,7]. Since metastases are responsible for most cancer deaths in solid tumors, understanding the functional differences between tumor-derived and non-tumor-derived exosomes is an area ripe for investigation.

In addition, our scant knowledge of exosomes in the context of human immunodeficiency virus (HIV) infection severely limits our understanding of the role of exosomes in prominent non-AIDS-defining cancers (NADCs) such as lung cancer [8,9]. It is well documented that exosomes from a wide range of cells can transform the microenvironment by inducing pro- or anti-viral responses [10,11,12,13,14,15]. Moreover, HIV-infected cells facilitate viral spread through the modulation of EV generally, and exosomes, specifically [8].

Recently, the role of exosomes in HIV treatment has attracted interest [16]. Chen et al. reported that the HIV transactivation response (TAR) element RNA, as cargo in exosomes derived from HIV-infected T cells, promotes head and neck squamous cell carcinomas as well as lung cancer in vitro and in vivo (xenograft animal models) [9]. In addition, the absolute numbers of EV and the exosome specific marker CD9 protein, are increased in HIV-positive compared to HIV-negative subjects [17].

The aim of this study was to assess the hypothesis that plasma exosome concentrations are influenced by lung cancer, chemotherapy, and HIV-related immunodeficiency, and to evaluate their prognostic value. For these reasons, we quantified CD9-positive plasma exosomes in the peripheral blood of patients who had cancer or benign disease with different degrees of immunosuppression including healthy controls.

## 2. Materials and Methods

### 2.1. Study Design, Population and Data Collection

All patients enrolled in the current study were ≥18 years old and medically managed at either Johns Hopkins Hospital (JHU) or Mary Washington Hospital (MWH) according to current treatment guidelines and to disease stage, comorbidities, and performance status. Excluded were patients without histological confirmation of their diagnosis or who lacked complete clinical records. A written, informed consent was obtained from all participants, and the study was approved by both institutions’ review boards. Surgical patients were referred to the thoracic surgical service at the JHU or MWH between 2009 and 2011 for surgical resection with curative intent of a suspected lung cancer (squamous carcinoma, adenocarcinoma, adenosquamous or small-cell carcinoma). Approximately 25% had malignancy histologically confirmed preoperatively with the remainder being determined by pathological analysis of the surgical specimen. In total, we evaluated the concentration of CD9-positive overall plasma exosomes in 166 lung cancer patients from JHU (126 patients) and MWH (40 patients), respectively. Furthermore, plasma exosome concentrations were also evaluated in 20 lung granuloma patients, who presented during the same time period for suspected cancer, but pathologically had a histologically confirmed benign disease. Forty-four NSCLC (Non-small-cell lung carcinoma) patients who presented to the Johns Hopkins Medical Oncology Service with progressive metastatic disease after systematic chemotherapy were also included.

In addition, plasma samples from HIV seropositive patients with or without lung neoplasms, who had been recruited and followed in the context of a CT screening program, matched by age to HIV-negative patients, were also included in order to clarify if immunological background can influence plasma exosome levels. In particular, CD9-positive exosomes were measured in 31 HIV+/lung cancer– (HIV+/LC–), 3 HIV+/Granuloma (HIV+/Gra) and 19 HIV+/lung cancer+ (HIV+/LC+) patients. Finally, samples from healthy controls and related demographic data were retrieved from the Healthy Control Biobank of the Molecular Oncology Laboratory at the University of Patras, Greece [18]. CD9 plasma-derived exosomes were isolated from 49 healthy controls. Nine healthy controls (18%) were smokers (Table 1).

Pathological and demographic data, treatment management and 5-year survival outcomes were obtained from the medical records, pathology reports or through public notifications. Staging of the lung cancer patients was determined based on the 7th edition of the AJCC Staging System [19]. In all, CD9 plasma-derived exosomes were isolated from 49 healthy controls, 20 patients with granulomas, 166 lung cancer patients, 44 metastatic NSCLC patients who progressed on chemotherapy, 31 HIV+/LC–, 3 HIV+/Gra and 19 HIV+/LC+ patients. Relevant clinicopathological information and specific clinicopathological characteristics of our study population are presented in Table 1 and Table 2.

### 2.2. Collection of Plasma Samples, Exosomes Isolation, and Quantification

To obtain plasma samples, EDTA-treated blood samples were collected, and after 2 preparatory spins at 3000 rpm for 3 min, were immediately stored at −80 °C until analysis. Exosomes were purified from plasma samples by using a commercially available kit (ExoTEST, HansaBioMed Life Sciences Ltd., Tallinn, Estonia), which is based on the double-sandwich ELISA technique, according to the manufacturer’s instructions as briefly described. The plasma samples were thawed at room temperature for approximately 1 h and then further processed by centrifugation in order to remove residual red blood cells and other cellular remnants. The conditions of the 3 centrifugation steps were: 10 min at 300× *g*, 20 min at 120× *g*, and 30 min at 10,000× *g*. After each step, the supernatants were transferred to new tubes and the pellets were discarded. Then, 100 µL of plasma was added to each well of a CD-9 pre-coated 96-well plate and incubated initially at room temperature for 30 min and then at 4 °C overnight. After 3 washes with the provided washing buffer, 100 µL of anti-CD9 Mab (diluted to 2 µg/mL) were added in each well and incubated initially for 15 min at room temperature and subsequently for 2 h at 4 °C. The 3 washing steps were repeated, 100 µL of HRP-conjugated anti-mouse antibody (dilution 1:2000) was added, and the plates were incubated for 15 min at room temperature while shaking. Then, the plates were incubated for 1 h at 4 °C. After 3 additional washing steps, 100 μL of Substrate Chromogenic Solution was added to each well and the plates were incubated at room temperature in the dark for 5–10 min. The reaction was blocked by adding 100 μL of stop solution to each well and optical densities (ODs) were measured at 560 nm and subtracted from the absorbance at 450 nm. The absolute quantification of isolated exosomes was conducted using the standard curve method. For each plate, a standard curve was created based on the ODs of the standards of known exosome concentrations provided in the kit. These were run in duplicate and a mean of each set of duplicate standards was used.

### 2.3. Verification of Isolated Plasma Exosomes

Transmission electron microscopy (TEM) was utilized to verify our methodological approach and was performed at the Microscope Facility at the Johns Hopkins University School of Medicine. Representative samples with low, intermediate, and high optical densities (ODs) as well as standard exosomes provided with the kit, were assessed by TEM analysis immediately after the completion of the quantification process.

Samples were fixed in 4% paraformaldehyde (freshly prepared from EM grade Prill form (Electron Microscopy Sciences (EMS)), Hatfield, PA, USA) with 1.5% glutaraldehyde (EMS, Hatfield, PA, USA), 3 mM MgCl2 (Polysciences, Inc., Warrington, PA, USA), in a 0.1 M sodium cacodylate buffer (EMS, Hatfield, PA, USA), and at a pH 7.2 for 1 h at room temperature. After a buffer rinse, the samples were postfixed in 1% osmium tetroxide (EMS, Hatfield, PA, USA) mixed with 0.8% potassium ferrocyanide (EMS, Hatfield, PA, USA) in a 0.1 M sodium cacodylate buffer (1 h) on ice in the dark. Following a 0.1 M maleate buffer (ThermoFisher Scientific, Fair Lawn, NJ, USA) rinse, the samples were stained enbloc with 2% uranyl acetate (Polysciences, Inc., Warrington, PA, USA) (0.22 µm filtered, 1 h in the dark) and maleate buffer, dehydrated in a graded series of ethanol (Polysciences, Inc., Warrington, PA, USA), and embedded in Eponate 12 (Ted Pella Inc., Redding, CA, USA) resin. Samples were polymerized at 37 °C for 2–3 days and then at 60 °C overnight. Thin sections, 60 to 90 nm, were cut with a diamond knife on the Reichert-Jung Ultracut E ultramicrotome (Leica Microsystems, Vienna, Austria) and handled with 2 × 1 mm formvar-coated copper slot grids (EMS, Hatfield, PA, USA). Samples were imaged on a Philips CM120 (ThermoFisher Scientific, Fair Lawn, NJ, USA) at 80 kV. Images were captured with an AMT XR80 CCD (8 megapixel) camera (AMT Direct Imaging, Woburn, MA, USA).

Furthermore, after overnight incubation, detached isolated exosomes were also examined by TEM prior to their quantification. A solution of 0.01% Triton (ThermoFisher Scientific, Fair Lawn, NJ, USA) 100× for 5 min with gentle scraping was used to detach the isolated exosomes. Exosome samples (10 μL) were adsorbed to glow discharged carbon-coated 400 mesh copper grids for 5 min before TBS (ThermoFisher Scientific, Fair Lawn, NJ, USA) (Tris Buffered Saline) rinse, followed by 1% PTA (MilliporeSigma, Burlington, MA, USA) (phosphotungstic acid). Grids were imaged on a Philips CM120 at 80 kV equipped with an AMT XR80 CCD (8 megapixel) camera. In addition, as a negative control, we used the supernatant from a well after incubation with PBS and the detachment procedure is as described for the remaining samples.

### 2.4. Statistical Analysis

All data were statistically analyzed using the IBM SPSS Statistics software for Windows, Version 21.0 (IBM Corp, Armonk, NY, USA). For all comparisons, statistical significance was defined as *p* < 0.05 and all statistical tests were two-sided. Differences between exosome concentrations, the categorical pathological parameters of the tumors, and the clinical characteristics of the patients were evaluated with the Kruskal-Wallis test followed by pairwise comparisons using the Mann-Whitney *U* test with a Bonferroni correction (a = 0.05 divided by the number of comparisons) for ordinal variables, χ^2^ test for nominal variables, and with Spearman correlations for comparisons between continuous variables. The X-tile software was used to provide the best cut-off point [20].

The time from the day of surgery or from the day of histological or cytological diagnosis until the day of the death or until the day of the last follow-up—if patients remained alive—was used for the calculation of overall survival (OS). The Kaplan-Meier method was used for univariate analysis and estimation of survival rates, and the log-rank test was used to determine the statistical significance. Cox proportional hazards models were used for multivariate analysis of the studied parameters in correlation with OS.

## 3. Results

### 3.1. Verification of Isolated Exosomes

**Figure 1:**
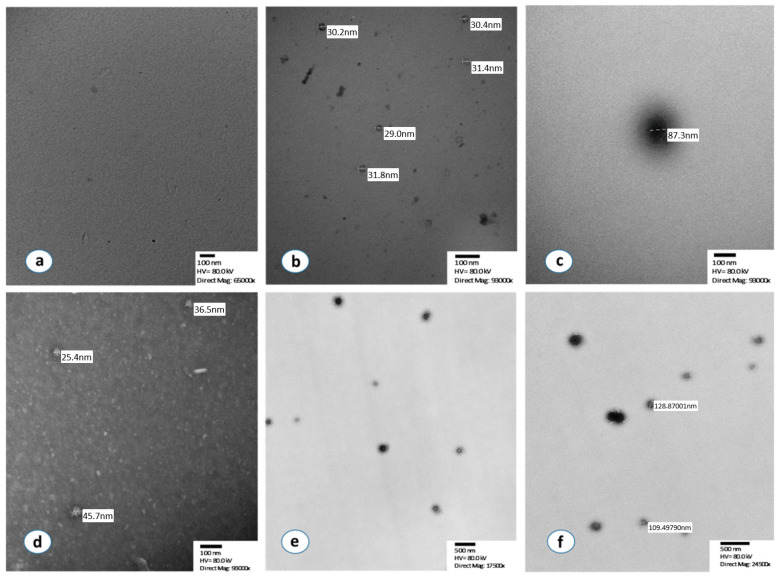
Morphological characterization of isolated exosomes by using transmission electron microscopy. (**a**) Negative control after negative staining, (**b,c**) Standard normal exosomes, supplied in the ExoTEST kit, after the immunoreaction with chromogen and without staining, (**d**) Isolated and detached exosomes from a NSCLC patient with negative staining, (**e**) Isolated exosomes from a lung cancer patient after immunoreaction with chromogen without staining, (**f**) Sample, isolated exosomes, after immunoreaction with chromogen, no staining.

In the first step of the analysis, we verified using TEM that the source of the detected signal was isolated exosomes (Figure 1). In particular, the vast majority of isolated extracellular vesicles had a maximum diameter between 30 and 150 nm, which is compatible with the specific characteristics of exosomes. TEM detection of exosomes was successfully performed before and after the immunoreaction with chromogen, both in the standard normal exosome samples (HansaBioMed Life Sciences Ltd., Tallinn, Estonia) as well as in isolated exosomes from our plasma samples. No signal was detected by TEM in the negative controls (Figure 1a–f).

### 3.2. Plasma Exosome Concentrations Differ among Subpopulations

**Figure 2:**
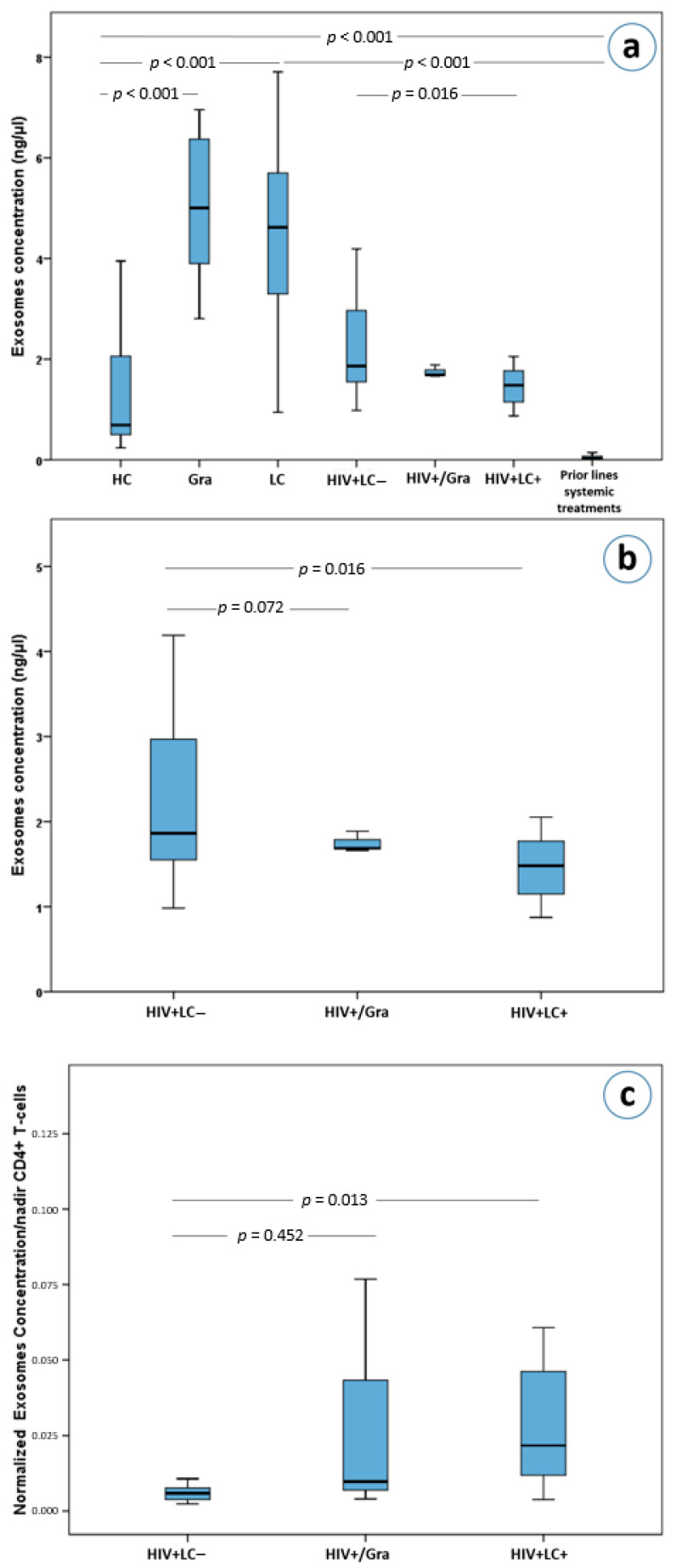
(**a**) Exosome concentrations in lung cancer and HIV patients as well as in healthy controls, (**b**) exosome concentrations in HIV+/LC–, HIV+/Gra and HIV+/LC+ patients, (**c**) normalized exosome concentrations based on nadirs of CD4(+) T-cells in HIV+/LC–, HIV+/Gra and HIV+/LC+ patients. Abbreviations: HC; Healthy controls, Gra; granulomas, LC; lung cancer patients, HIV+LC–; HIV seropositive patients without LC, HIV+/Gra; HIV seropositive patients with Gra, HIV+LC+; HIV seropositive patients with LC.

The median plasma exosome concentrations varied significantly among the seven subgroups analyzed (χ^2^ = 183.3, df = 6, Kruskal-Wallis *p* < 0.0001). After a Bonferroni adjustment for 21 comparisons, plasma exosome concentrations were found to differ between healthy controls and immunocompetent patients with granulomas (Mann-Whitney U test with Bonferroni correction, a = 0.0024, *p* < 0.001, Figure 2a) as well as between healthy controls and patients with lung cancer (*p* < 0.001, Figure 2a). Surprisingly, exosome plasma concentrations in metastatic lung cancer patients who progressed on chemotherapy were much lower compared to both healthy controls (*p* < 0.001, Figure 2a), as well as other lung cancer patients (*p* < 0.001, Figure 2a). In addition, these treated lung cancer patients had lower plasma exosome concentrations compared to patients with lung granulomas (*p* < 0.001, Figure 2a).

Interestingly, HIV+/LC– patients had higher exosome concentrations than HIV+/LC+ patients (*p* = 0.016, Figure 2a,b). However, HIV+/LC– patients’ exosome concentrations were not statistically significantly higher than that of HIV+/Gra patients (*p* = 0.72, Figure 2b). Furthermore, HIV-induced immunodeficient patients (HIV+/LC–, HIV+/Gra, HIV+/LC+) had lower plasma exosome concentrations compared to immunocompetent granuloma and lung cancer patients (*p* < 0.001) for all comparisons with the exception of comparing HIV+/Gra with lung cancer patients. The latter did not reach statistical significance upon Bonferroni adjustment, (*p* = 0.031, a = 0.0024, Figure 2a). Importantly, HIV+/LC– patients had significantly higher plasma exosome concentrations compared to healthy controls (*p* < 0.001, Figure 2a). More interestingly, HIV seropositive patients (HIV+/LC–, HIV+/Gra, HIV+/LC+) had higher exosome concentrations than lung cancer patients who received systemic therapy (*p* < 0.001 for all comparisons), while the difference between HIV+/Gra patients and treated lung cancer patients did not reach statistical significance (*p* = 0.006, a = 0.0024 after correcting for multiple testing, Figure 2a).

### 3.3. Normalized Exosomes Were Higher in HIV+/LC+ Patients

As already mentioned, the concentration of plasma exosomes in HIV+/LC– patients was higher than in HIV+/LC+ patients (*p* = 0.016, Figure 2b). On the other hand, no difference was observed between the HIV+/Gra and HIV+/LC– subgroups (*p* = 0.72, Figure 2b). Surprisingly, using the ratio of exosome concentrations over the nadir number of CD4(+) cells, we observed that HIV+/LC+ patients had significantly higher ratios than HIV+/LC– patients (Mann-Whitney U test with Bonferroni correction, a = 0.017 for 3 comparisons, *p* = 0.013, Figure 2c). This difference remained statistically significant in a multivariate analysis which included age, gender, CD4(+) T cells, white blood cells and race as cofactors (*p* < 0.001, adjusted R square = 0.962).

### 3.4. Plasma Exosomes, Disease Stage, and Gender

Despite the fact that no difference was detected between the concentrations of plasma exosomes of untreated lung cancer patients in different disease stages (Kruskal-Wallis *p* = 0.98), a statistically significant difference was observed between patients of each stage and healthy controls. In particular, stage I–IV lung cancer patients had significantly higher exosome concentrations than healthy controls after a Bonferroni adjustment for ten comparisons (Mann-Whitney U test with Bonferroni correction, a = 0.005, *p* < 0.001 for stages I–III and *p* = 0.011 for stage IV). On the other hand, no difference was observed in exosome concentrations between lung cancer stages in HIV-positive patients (Kruskal-Wallis *p* = 0.73).

### 3.5. Small Cell Carcinomas Have Higher Concentrations of Plasma Exosomes Than NSCLC

**Figure 3:**
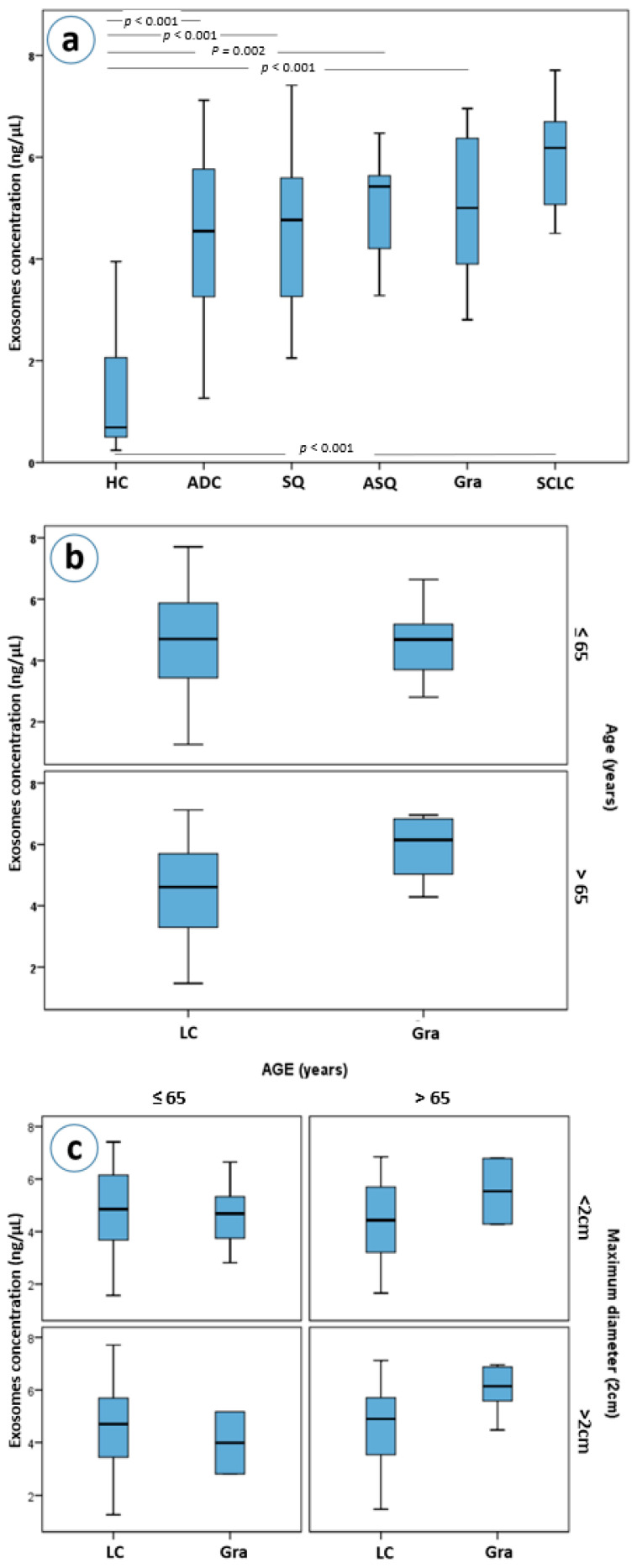
(**a**) Exosome concentrations by lung cancer histological type, (**b**) exosome concentrations in lung cancer and granuloma patients stratified by age, (**c**) exosome concentrations in lung cancer and granuloma patients with stratification based on age and maximum diameter of the mass. Abbreviations: HC; Healthy controls, ADC; adenocarcinoma, SQ; squamous, ASQ; adenosquamous, Gra; granulomas, SCLC; small-cell lung carcinomas, LC; lung cancer patients.

Exosome concentrations differ between all different pulmonary histologies and healthy controls (Kruskal-Wallis *p* < 0.001 for all histological subtypes, Figure 3a). Even though, patients with granulomas, as well as those with adenocarcinomas, squamous carcinomas, adenosquamous carcinomas and SCLC had higher plasma exosome concentrations than healthy controls, only in patients with granulomas and in those with SCLC were those differences statistically significant after a Bonferroni adjustment for 15 comparisons (both Mann-Whitney *p*’s < 0.001, a = 0.003, Figure 3a). Moreover, granuloma and SCLC patients had higher exosome concentrations compared to NSCLC patients as a group; a difference that also remained statistically significant after adjustment for multiple testing (Mann-Whitney *p* = 0.004 and *p* = 0.005, respectively).

### 3.6. Granulomas vs. Lung Cancer

Further stratification, based on the size and age of the patients, led to an interesting observation. Patients with granulomas over the age of 65 years were found to have higher concentrations of exosomes than patients with lung cancer of the same age (Mann-Whitney *p* = 0.021, Figure 3b). This difference was marginally greater when lesion size was considered, with patients with granulomas larger than 2 cm having higher plasma exosome concentrations than those of lung cancer patients (*p* = 0.045, Figure 3c).

### 3.7. Concentration of Plasma Exosomes Is Strongly Associated with Overall Survival (OS)

**Figure 4:**
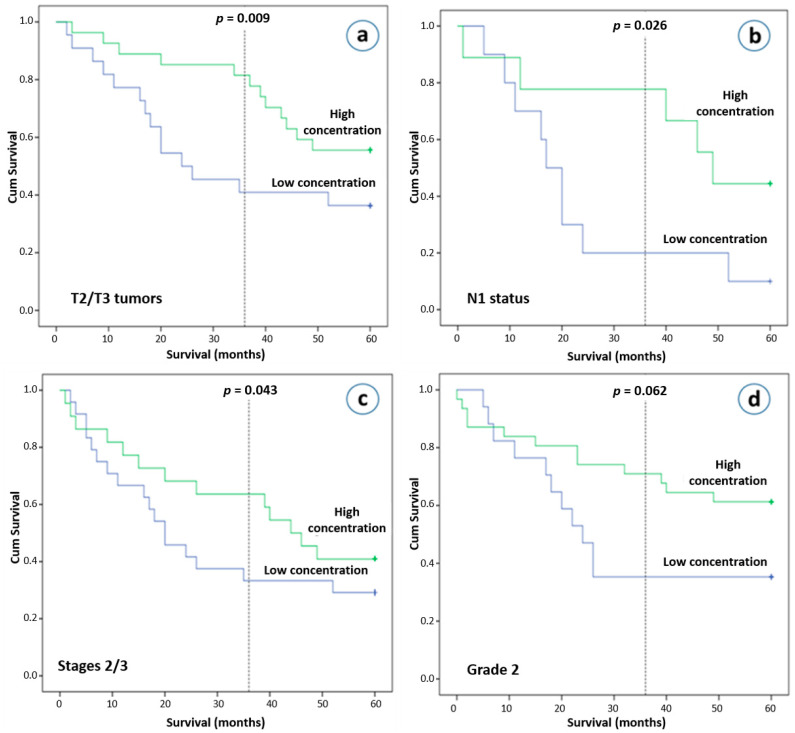
Overall survival (OS) and plasma exosome concentrations. Kaplan-Meier plot of 5-year OS according to the plasma exosome concentration (more than 4.71 ng/μL vs. less than 4.71 ng/μL). (**a**) Patients with T2 and T3 tumors, (**b**) patients with N1 disease, (**c**) patients with stage II and III, (**d**) patients with grade 2 disease.

Our final analysis investigated the significance of plasma exosomes in relation to clinical outcome. Although total plasma exosomes concentration was not found to be associated with overall survival (OS) in HIV seronegative NSCLC patients (*p* = 0.520), those who had T2 or T3 tumors as well as higher exosome concentrations were found to have better clinical outcomes after 3 years (*p* = 0.009), but not after 5 years of follow-up (*p* = 0.074, Figure 4a). NSCLC patients with N1 disease and higher exosome concentrations had improved clinical outcomes after 3- and 5-years’ observation (*p* = 0.026 and *p* = 0.050, respectively, Figure 4b). In addition, on univariate analysis, a higher exosome concentration was also associated with better survival in stage II and III NSCLC patients after the first 36 months (*p* = 0.044), but not after 5 years of follow-up (*p* = 0.326, Figure 4c). After the first few months of follow-up, an early and sustained separation of Kaplan-Meier curves was observed. We subsequently performed a multivariate analysis using a Cox proportional hazards regression analysis at 3 years, incorporating as covariates age, gender, stage, race. A high plasma exosome concentration was found to be an independent risk factor for better survival with a hazard ratio of 0.407 (95% confidence interval, 0.172–0.961; *p* = 0.040). Other interesting observations, although they did not reach statistical significance, were that NSCLC patients with pathological infiltration of lymphatics and higher exosome concentrations had better survival outcome (*p* = 0.132 after a 5-year follow-up), as well as that patients with intermediate differentiation and lower exosome concentrations had worse 3-year and 5-year overall survival rates (*p* = 0.056 and *p* = 0.122, respectively, Figure 4d).

### 3.8. No Association with Age, BMI, Maximum Diameter, Vessel Infiltration, Recurrence, Smoking Status, Grade and Race

No associations were observed in the plasma exosomes of HIV-negative lung cancer patients with age (Spearman, *p* = 0.602), BMI (Spearman, *p* = 0.513), white blood cell counts (Spearman, *p* = 0.221), platelets count (Spearman, *p* = 0.102), height (Spearman, *p* = 0.624), weight (Spearman, *p* = 0.2107), maximum diameter (Spearman, *p* = 0.750), recurrence (Mann-Whitney U Test, *p* = 0.476), smoking (Kruskal-Wallis, *p* = 0.710), grade (Kruskal-Wallis, *p* = 0.810), or vessel infiltration (Kruskal-Wallis, *p* = 0.674). Furthermore, plasma exosome concentrations were similar in patients of different races (Kruskal-Wallis, *p* = 0.835).

## 4. Discussion

In this study, overall plasma CD9-positive exosome concentrations were evaluated in subpopulations with different health backgrounds and different degrees of immunosuppression. The data from this study convincingly show that plasma exosome concentration in patients is altered depending on medical history and that cancer, HIV immunopositivity, as well as systemically administered chemotherapy can influence the levels of CD9-posistive plasma exosomes.

One of the most interesting findings of our study was that total plasma exosome concentration was found to be lower in healthy controls compared to immunocompetent patients with granulomas and compared to lung cancer patients. These results are consistent with previous studies [21,22,23]. In particular, Silva et al. have shown that the proportion of EpCAM-positive exosome levels in colorectal cancer patients was statistically higher than those of healthy controls [22]. In addition, the mean plasma exosome level in ovarian cancer patients was also higher compared to that of healthy controls [23]. The same group has shown that benign tumors of the ovaries have significantly increased amounts of plasma exosomes compared to healthy controls [23]. These findings further support our notion that neoplasms may influence the exosome concentration in plasma.

Another very important finding of our study was that plasma exosome concentrations did not correlate with stage and that the association of plasma exosome concentrations was an independent prognostic factor for OS. Higher exosome concentrations were related to better survival in stages II and III NSCLC patients. Consistent with our findings, Matsumoto et al. have shown that plasma-derived exosomes in esophageal squamous cell carcinoma patients were not associated with clinical stage and were an independent prognostic factor for OS with lower levels of exosomes being associated with poor prognosis [21]. Similarly, in a small cohort of 91 colorectal cancer patients, plasma exosomes were not associated with clinical stage. On the other hand, higher levels of plasma exosomes before surgery in the same patients tended to be associated with shorter OS compared to patients with low levels (*p* = 0.056), but not with disease-free survival [22]. Similarly, Liu et al. recently reported that high plasma exosome levels are associated with a poor clinical outcome of NSCLC patients as well as that NSCLC patients with stages IIIb–IV had higher plasma exosomes compared to stages I–IIIa patients [24]. This study by Liu et al. is inconsistent with our results. This discordance may reflect different methodological approaches. Our study is based on the detection of exosomes using an immunoaffinity-based method and a CD9 specific marker, the expression of which in lung cancer has recently been confirmed in all patients (100%) by Sandfeld-Paulsen et al. [25]. On the other hand, although there is not a reliable technique for isolating and quantifying exosomes, the acetylcholinesterase (AChE) method used by Liu et al. raises many concerns, since AChE is not a specific exosomal marker. In addition, as Liu et al. discussed, AChE activity may be influenced by contaminants which can be co-isolated with their precipitation technique [24].

Our study further revealed that plasma-derived exosome concentrations in lung cancer patients who had received chemotherapy were much lower compared to healthy controls and untreated lung cancer patients. In line with our results, Hong et al. have shown that acute myeloid leukemia (AML) patients who were treated with chemotherapy had a significant reduction in the exosomal protein concentration 14 days after chemotherapy initiation [26]. Similarly, head and neck cancer patients upon systemic therapies had decreased levels of exosomal proteins [27]. Moreover, Szajnik et al. have documented that exosomal protein levels variably change after chemotherapy administration in ovarian cancer patients, with some patients having dramatic reductions in exosomal protein levels [23]. In addition, Muller et al. documented that protein levels of exosomes in glioma patients who had received antitumor vaccines were significantly decreased post vaccination [28].

Our study also showed that HIV+/ LC– patients had higher exosome concentrations than HIV+/LC+ patients. In addition, both of these patient subgroups had statistically significant higher concentrations of plasma exosomes compared to healthy controls, but lower concentrations compared to HIV-/LC+ patients. Unfortunately, data on plasma exosomes in lung cancer patients, especially in HIV-positive patients, are extremely limited. However, our observation is in concordance with the observation of Hubert et al. that plasma-derived exosome concentrations (AChE+) of treatment-naïve HIV-1-infected patients are higher compared to uninfected control subjects [29]. That difference in exosome concentration between HIV patients and healthy controls is also supported by Jeannin et al. [30]. Their patients who were infected with human T-lymphotropic retrovirus type 1 (HTLV-1), an ancient virus similar to HIV, had abundant extracellular vesicles concentrations that were significantly higher than non-infected individuals [30].

Another intriguing finding of the present study is the correlation of normalized exosome concentrations with the presence of lung cancer in HIV+ patients. Surprisingly, we observed that normalized plasma-derived exosomes were higher in HIV+/LC+ than HIV+/LC– patients. Although this normalization could be characterized as arbitrary, it may reflect a tight molecular connection between exosome levels and immune status. Specifically, Hubert et al. have shown that an abundance of exosomes is negatively associated with CD4(+) T-cell count, with CD4(+) T-cell nadir being a good predictor of immune system activation [29]. In addition, it is well documented that HIV-1 replication in primary CD4(+) T lymphocytes is permitted under the influence of HIV-1-infected cells derived exosomes [10]. Furthermore, the possible inverse relation between exosomes and CD4(+) T cells is supported by Lenassi et al., who documented that HIV-related exosomes facilitate the apoptosis of CD4(+) T cells [31].

A major limitation of our study is the number of HIV-positive patients, especially those with lung cancer, which do not permit the survival analysis in this subpopulation as well as the co-estimation with other parameters such as the counts of CD4(+) and CD(8)+ T cells. In addition, stage IV lung cancer patients are not equally represented in our cohort since many of our blood samples were from the lung cancer plasma biobank that contains a higher percentage of surgical patients. Furthermore, a two-stage design incorporating a validation group would offer more robust results and definitive conclusions. In addition, differences in the cargo of exosomes among the different subpopulations were not studied as aims of this study. Heterogeneity of the cohorts in regard to age and race could be a matter of major concern, as HIV-positive patients were mainly younger and almost all African Americans. However, Eitan et al. have reported that race does not influence concentrations of extracellular vesicles, and that advancing age is associated with decreasing EV concentration in the general population [32]. In our study, no association was observed between exosome concentrations and age or race, either in HIV-positive or HIV-negative lung cancer patients. Furthermore, it would be desirable to have enrolled in this study patients with advanced NSCLC treated with immunotherapy, given its effectiveness in this population, as well as to have investigated the potent relation of the tumor immune microenvironment with the levels of plasma exosomes.

## 5. Conclusions

In summary, we report for the first time that CD9-positive plasma exosomes are increased in HIV seropositive and HIV seronegative lung cancer patients compared to healthy controls, while chemotherapy-treated lung cancer patients have lower plasma exosome levels. This study also shows that in chemotherapy-naïve patients, plasma exosome levels are directly correlated with prognosis with higher concentrations associated with longer OS. These findings further support previous literature on the translational significance of total plasma exosomes in cancer patients, despite different immunological contexts.

## Figures and Tables

**Table 1:** Demographic characteristics of patients and healthy controls.

Demographic Characteristics	LC Patients *n* (%)	Granulomas Patients *n* (%)	Pre-Treated LC Patients *n* (%)	HIV+/LC– Patients *n* (%)	HIV+/Gra *n* (%)	HIV+/LC+ Patients *n* (%)	Healthy Controls *n* (%)
Total	166 (100)	20 (100)	44 (100)	31 (100)	3 (100)	19 (100)	49 (100)
Age (years) Median (range)	70 (44–90)	62 (35–82)	64 (46–82)	51 (42–67)	47 (46–49)	54 (46–83)	57 (36–90)
Gender							
Total	166 (100)	20 (100)	44 (100)	31 (100)	3 (100)	19 (100)	49 (100)
Male	80 (48.2)	11 (55.0)	23 (52.3)	21 (67.7)	3 (100)	11 (57.9)	18 (36.7)
Female	86 (51.8)	9 (45.0)	21 (47.7)	10 (32.3)	-	8 (42.1)	31 (63.3)
Race							
Total	166 (100)	20 (100)	44 (100)	31 (100)	3 (100)	19 (100)	49 (100)
White	120 (72.3)	17 (85.0)	34 (77.2)	4 (12.9)	-	1 (5.3)	43 (87.8)
African American	29 (17.5)	1 (5.0)	9 (20.5)	27 (87.1)	3 (100)	17 (89.4)	2 (4.1)
Asian	4 (2.4)	0 (0)	-	-	-	1 (5.3)	4 (8.1)
Other	6 (3.6)	2 (10.0)	1 (2.3)	-	-	-	-
NA	7 (4.2)	-	-	-	-	-	-
Smoking (pack-years)							
Total	166 (100)	20 (100)	44 (100)	31 (100)	3 (100)	19 (100)	49 (100) *
Cases (%)	155 (93.4)	20 (100)	40 (90.9)	25 (80.6)	2 (66.7)	13 (68.4)	9 (18.4)
Mean (range)	48.2 (0–240)	21.35 (0–60)	36.9 (0–124)	29.76 (3–60)	38 (6–70)	35 (10–75)	20.5 (5–36)
NA	11 (6.6)	-	4 (9.1)	6 (19.4)	1 (33.3)	6 (31.6)	7 (14.3)

Abbreviations: LC, lung cancer; HIV+/LC–, HIV seropositive patients without lung neoplasms; HIV+/Gra, HIV seropositive patients with lung granulomas; HIV+/LC+, HIV seropositive patients with LC; NA, data not available or unknown; *n* (%), number of cases as a percentage. * Forty healthy controls were non-smokers.

**Table 2:** Clinicopathological characteristics and survival outcome in patient subgroups of our study. Abbreviations: LC, lung cancer; HIV+/LC–, HIV seropositive patients without lung neoplasms; HIV+/Gra, HIV seropositive patients with lung granulomas; HIV+/LC+, HIV seropositive patients with LC; min, minimum; max, maximum; NA, data not available or unknown, *n* (%), number of cases in percentage.

Clinicopathological Characteristics	LC Patients *n* (%)	Granuloma Patients *n* (%)	Pre-Treated LC Patients *n* (%)	HIV+/Gra Patients *n* (%)	HIV+/LC+ Patients *n* (%)
Total	166 (100)	20 (100)	44 (100)	3 (100)	19 (100)
Primary location					
Total	166 (100)	20 (100)	44 (100)	3 (100)	19 (100)
Left lung	56 (33.7)	4 (20)	-	2 (66.7)	6 (31.6)
Right lung	70 (42.2)	6 (30)	-	1 (33.3)	12 (63.2)
NA	40 (24.1)	10 (50)	44 (100)	-	1 (5.3)
Histology					
Total	166 (100)	20 (100)	44 (100)	3 (100)	19 (100)
Squamous	58 (35.0)	-	12 (27.3)	-	5 (26.3)
Adenocarcinoma	91 (54.8)	-	31 (70.5)	-	12 (63.2)
Adenosquamous	8 (4.8)	-	1 (2.3)	-	-
Small-cell carcinoma	9 (5.4)	-	-	-	2 (10.5)
NA	-	-	-	-	-
Stage					
Total	166 (100)	20 (100)	44 (100)	3 (100)	19 (100)
I	68 (41.0)	-	-	-	9 (47.4)
II	47 (28.3)	-	-	-	4 (21)
III	45 (27.1)	-	-	-	4 (21)
IV	6 (3.6)	-	44 (100)	-	1 (5.3)
NA	0 (0.0)	-	-	-	1 (5.3)
Grade					
Total	166 (100)	20 (100)	44 (100)	3 (100)	19 (100)
I	5 (3.0)	-	-	-	1 (5.3)
II	66 (39.8)	-	-	-	2 (10.5)
III	50 (30.1)	-	-	-	4 (21.1)
NA	45 (27.1)	-	44 (100)	-	12 (63.1)
Maximum diameter (cm)					
Total	166 (100)	20 (100)	44 (100)	3 (100)	19 (100)
Cases (%)	166 (100)	20 (100)	-	3 (100)	17 (89.5)
Mean (range)	3.36 (0.80–13.00)	1.99 (0.50–8.00)	-	1.3 (1.10–1.50)	2.85 (0.90–6.00)
NA	-	-	44 (100)	-	2 (10.5)
Lymph node infiltration					
Total	166 (100)	20 (100)	44 (100)	3 (100)	19 (100)
No	93 (56.0)	-	-	-	11 (57.9)
Yes	71 (42.8)	-	-	-	7 (36.8)
NA	2 (1.2)	-	44 (100)	-	1 (5.3)
Survival (3 years)					
Total	166 (100)	20 (100)	44 (100)	3 (100)	19 (100)
Dead	40 (24.1)	1(0.05)	40 (90.9)	-	-
Alive	87 (52.4)	2 (0.1)	4 (9.1)	-	-
NA	39 (23.5)	17 (85)	-	3 (100)	19 (100)
Survival (5 years)					
Total	166 (100)	20 (100)	44 (100)	3 (100)	19 (100)
Dead	52 (31.3)	1 (0.05)	43 (97.7)	-	-
Alive	56 (33.7)	1 (0.05)	1 (3.3)	-	-
NA	58 (35.0)	18 (90)	-	3 (100)	19 (100)

## Data Availability

The raw data that support the findings of this study are available from the corresponding author upon reasonable request.
